# 318. A Pain in the Neck: Cervical Pyomyositis, a Rare Case of Extraintestinal Nontyphoidal Salmonellosis Further Characterized by Whole-Genome Sequencing.

**DOI:** 10.1093/ofid/ofac492.396

**Published:** 2022-12-15

**Authors:** Pishoy Haroun, Shangxin Yang, Paul C Adamson, Ashlyn N Sakona

**Affiliations:** Univeristy of California, Los Angeles, Los Angeles, California, USA, Los Angeles, California; Univeristy of California, Los Angeles, Los Angeles, California, USA, Los Angeles, California; UCLA School of Medicine, Los Angeles, California; Univeristy of California, Los Angeles, Los Angeles, California, USA, Los Angeles, California

## Abstract

**Background:**

Nontyphoidal salmonellosis (NTS) is the second-most common foodborne illness in the US, but extraintestinal manifestations are rare. We describe a case of sternocleidomastoid (SCM) pyomyositis, a rare entity, caused by *Salmonella enterica,* further delineated by whole-genome sequencing (WGS).

A 55 year old male with liver cirrhosis and uncontrolled type-II diabetes mellitus presented with a six-day history of an enlarging left-sided neck mass. He had no fevers, chills, night sweats, nausea, vomiting or diarrhea. He had recently returned from Saudia Arabia with exposures to camels, bats and lemurs. He did not consume raw foods or dairy products.

Physical exam revealed normal vital signs and a large firm neck mass. Labs were notable for leukocytosis and hyperglycemia. Neck computed tomography revealed a 6 cm heterogeneous mass inseparable from the left SCM. He required repeated drainage procedures; histopathology revealed skeletal muscle with inflammation, but no malignancy. Blood and procedural cultures grew Salmonella group B. He was treated with ceftriaxone then trimethoprim-sulfamethoxazole. Follow-up 4 weeks after presentation revealed only residual induration.

**Methods:**

WGS was performed using Illumina MiSeq. Genotype and antimicrobial resistance markers were identified using MLST, KmerFinder and ResFinder on Center of Genomic Epidemiology. Virulence factors were identified using VFanalyzer.

**Results:**

The bacteria was identified as *Salmonella enterica subspecies enterica* serovar Typhimurium and typed as ST19, the major phylogroup of Typhimurium globally. Characteristics associated with invasive NTS (iNTS) identified by WGS included genes encoding Type III secretion system and Invasin A. Other virulence factors included fimbrial adherence determinants, macrophage inducible genes, and an intracellular toxin SpvB. Resistance genes included aac(6')-Iaa, which confers resistance to tobramycin and amikacin, and qnrB19, which predicts resistance to ciprofloxacin. However, no quinolone resistance-related mutations were detected in gyrA, gyrB, parC and parE genes.

AMR Genes

**Figure:**
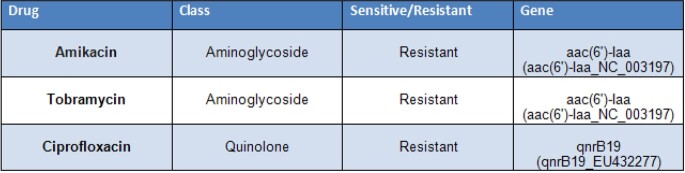

Antimicrobial resistance genes identified by whole-genome sequencing of the Salmonella isolate.

**Conclusion:**

Patients with uncontrolled diabetes are at increased risk of iNTS with or without associated diarrhea. WGS can identify additional factors that may influence morbidity and resistance.

**Disclosures:**

**All Authors**: No reported disclosures.

